# Inoculation with ASFV-Katanga-350 Partially Protects Pigs from Death during Subsequent Infection with Heterologous Type ASFV-Stavropol 01/08

**DOI:** 10.3390/v15020430

**Published:** 2023-02-03

**Authors:** Mikhail E. Vlasov, Irina P. Sindryakova, Dmitry A. Kudrjashov, Sergey Y. Morgunov, Olga L. Kolbasova, Valentina M. Lyska, Sergey P. Zhivoderov, Elena Y. Pivova, Vladimir M. Balyshev, Alexey D. Sereda, Denis V. Kolbasov

**Affiliations:** Federal Research Center for Virology and Microbiology (FRCVIM), Petushki District, Vladimir Region, 601125 Volginsky, Russia

**Keywords:** African swine fever virus, seroimmunotype, genotype, attenuated strains, heterologous protection

## Abstract

African swine fever virus (ASFV) is an extremely genetically and phenotypically heterogeneous pathogen. Previously, we have demonstrated that experimental inoculation of pigs with an attenuated strain, Katanga-350 (genotype I, seroimmunotype I) (ASFV-Katanga-350), can induce protective immunity in 80% of European domestic pigs against the homologous virulent European strain Lisbon-57. At least 50% of the surviving pigs received protection from subsequent intramuscular infection with a heterologous virulent strain, Stavropol 01/08 (genotype II, seroimmunotype VIII) (ASFV-Stavropol 01/08). In this study, we assessed clinical signs, the levels of viremia, viral DNA, anti-ASFV antibodies and post-mortem changes caused by subsequent intramuscular injection with ASFV-Katanga-350 and heterologous ASFV-Stavropol 01/08. Inoculation of pigs with the ASFV-Katanga-350 did not protect animals from the disease in the case of the subsequent challenged ASFV-Stavropol 01/08. However, 40% of pigs were protected from death. Moreover, the surviving animals showed no pathomorphological changes or the presence of an infectious virus in the organs after euthanasia at 35 days post challenging. The ability/inability of attenuated strains to form a certain level of protection against heterologous isolates needs a theoretical background and experimental confirmation.

## 1. Introduction

African swine fever (ASF) is a viral infectious disease affecting all breeds and age groups of animals of the *Suidae* family. The most widespread form of the disease is acute, characterized by fever, toxicosis and hemorrhagic diathesis with a lethality of up to 100%. Some countries of East Africa (endemic regions), Spain, Portugal, Dominican Republic, Estonia, Sardinia, and China reported a subacute form of the disease with a mortality of 30 to 70%, as well as a chronic one with very low mortality levels [1,2]. There is currently no fully licensed vaccine available, and research efforts to develop effective (live, inactivated or subunit) vaccines have not reached the intended effect [3]. The pigs who survive viral infection with moderately virulent or attenuated strains of ASF virus (ASFV) have protective immunity with resistance to reinfection (homologous, but rarely to heterologous) [4]. 

The use of attenuated strains is currently the most plausible approach to develop an effective ASF live vaccine (LAVs). The rational development of attenuated strains is through attenuation of viruses by passaging in cell cultures or genetic manipulation.

We have noticed the facts indicating the possibility of the formation of heterologic ASF protection. Cross-protection—the ability of a LAV to protect against viruses from heterologous geno- or serogroups, not just the homologous parental strain—is an important open question in ASF vaccine development. In 2017, Monteagudo et al., published data on BA71ΔCD2, a CD2v/EP402R genedeleted strain, demonstrating dose-dependent protection against parental BA71 and the heterologous genotype I E75 and genotype II Georgia 2007/1 ASFV strains, both belonging to the same clade (clade C); it was the first time that heterologous protection was fully achieved [5]. This group later first observed that 83.3% of the pigs immunized once with BA71∆CD2 survived the tick-bite challenge using *Ornithodoros* sp. soft ticks naturally infected with the RSA/11/2017 strain (genotype XIX, clade D). Second, only 33.3% survived the challenge with Ken06.Bus (genotype IX, clade A), which is phylogenetically more distant to BA71∆CD2 than the RSA/11/2017 strain. Homologous prime-boosting with BA71∆CD2 only improved the survival rate to 50% after Ken06.Bus challenge, all suffering mild ASF-compatible clinical signs, while 100% of the pigs immunized with BA71∆CD2 and boosted with the parental BA71 virulent strain survived the lethal challenge with Ken06.Bus, with almost no clinical signs of the disease [6].

The possibility of the induction of protective immunity in domestic pigs against two virulent African isolates of ASFV was demonstrated after experimental immunization with a non-virulent ASFV genotype I isolate from Portugal, OURT88/3, followed by a boost with a closely related virulent isolate, OURT88/1. In the experiments, animals were divided into several groups: one group 3 weeks after immunization was challenged with the West African isolate I of genotype Benin 97/1, and the other group was infected with the virulent isolate X of genotype Uganda 1965. Overall, 85.7% and 100% of pigs were protected against infection with Benin 97/1 and Uganda 1965 ASFV, respectively. More than 78% of pigs infected with Benin 97/1 and 50% of pigs infected with Uganda 1965 were completely protected, showing no signs of disease or development of viremia [7].

Previously, we demonstrated that experimental double inoculation of pigs with attenuated ASFV genotype I, seroimmunotype I ASFV-Katanga-350 can induce protective immunity in 80% of European domestic pigs against the homologous virulent European strain of ASFV, Lisbon-57. At least 50% of animals that survived were protected from subsequent challenge with a heterologous virulent European strain ASFV-Stavropol 01/08, belonging to genotype II and seroimmunotype VIII [8]. The next stage of our work was to establish the protective properties of the attenuated ASFV-Katanga-350 in relation to the virulent heterologous ASFV- Stavropol 01/08, bypassing the stage of infection of vaccinated pigs with the homologous virulent strain Lisbon-57. 

## 2. Materials and Methods

### 2.1. Viruses

The following ASFV strains were received from the collection of microorganisms of the Federal Research Center for Virology and Microbiology (FRCVIM, Volginsky, Russia): Stavropol 01/08, Katanga-350.

The strain Katanga-350 was obtained by multiple passaging of the original Katanga strain kindly provided by Dr. W. Plowright (1978) on primary pig bone marrow cells (PBMC).

### 2.2. Animal Experiments and Ethics Statement

Two- to three-month-old female and male (20–30 kg) pigs of the Large White pig breed purchased from the Experimental Animal Preparation Sector of the FRCVM were used in the experiment. They were housed in a BSL3-Ag laboratory of the FRCVM. There was a 5-day acclimation period before commencement of the study. During this period, pigs were trained to chew on the ropes used to collect oral fluid samples. The pigs were kept and euthanized in accordance with the AVMA Guidelines for the care and use of laboratory animals [9], and all efforts were made to minimize suffering. 

Twelve pigs were randomly divided into three groups housed in three separate premises. There were five pigs in group 1 and 2. Group 3 (control) consisted of two pigs. Pigs in group 1 (#1–5) were inoculated intramuscularly on day 0 with the ASFV-Katanga-350 at a dose of 10^6.00^ HAU_50_ (50% hemadsorbing units). Group 2 (pigs #6–10) were inoculated intramuscularly with the ASFV-Katanga-350 at a dose of 10^6.00^ HAU_50_ twice (on days 0 and day 14). Animals from groups #1–3 were challenged intramuscularly with the ASFV-Stavropol 01/08 at a dose of 10^3.00^ HAU_50_ on the 28th day.

Blood specimens from the anterior vena cava were sampled at 0, 3, 5, 7, 10, 14, 21, 28 days post inoculation (dpi) with the ASFV-Katanga-350 and at 3, 5, 7, 10, 14, 21, 28, 35 days post challenging (dpc) with the ASFV-Stavropol 01/08, and collected in test tubes (10 mL each) with a coagulant to receive the serum and with an anticoagulant (lithium heparin) to determine the viremia levels and tubes with EDTA for ASFV genome determination [10].

### 2.3. Preparation of Peripheral Blood Leukocytes Cell (PBLS) Culture

Heparinized blood samples collected from the anterior vena cava were layered on a Ficoll–Hypaque gradient (density 1.077 g/cm^3^, GE Healthcare, Chicago, IL, USA) and centrifuged (22 °C, 400× *g*, 30 min). Cells from the interphase were collected and washed out with Hanks balanced saline solution three times by centrifugation (4 °C, 400× *g*, 5 min). As a growth medium (pH 7.60–7.65), we used 0.1% lactalbumin hydrolysate in Earle’s saline solution with 10% donor pig blood serum. PBLS primary cell culture was distributed in 48-well plastic micropanels (Nunc, Roskilde, Denmark) with a working volume of 1.0 mL. Wells were filled with cell suspension to achieve a concentration of 3.0–3.5 million cells/mL. Micropanels were incubated in a CO_2_ incubator and under the following conditions: CO_2_ concentration 5%, relative humidity 90%, and temperature (37.0 ± 0.5) °C.

### 2.4. Sample Collection

To collect oral fluids, a cotton triple-stranded rope (TD PROMT LLC, Lipetsk, Russia) was suspended in a pen in front of each animal at shoulder height for 20–30 min for chewing. For this period of time the animals were kept individually. The pigs chewed on the ropes, and after that the oral fluids were collected individually by cutting off the wet end of the rope and putting it into a plastic bag (TD PROMT LLC, Lipetsk, Russia). Then the liquid was squeezed into a plastic vial (Fisher Scientific Company LLC, Pittsburgh, PA, USA) and centrifuged at 1200× *g* for 2 min. The supernatant was collected for testing. Sera and whole blood samples were obtained by jugular venipuncture. To obtain whole blood, the blood samples were collected in EDTA tubes (TD VIK LLC, Lyubertsy, Russia), and to obtain blood serum, the whole blood was collected in serum tubes (TD VIK LLC, Lyubertsy, Russia), allowed to clot, and then centrifuged for 10 min at 2000× *g*. Three aliquots of all the samples described above were prepared and stored in 5 mL or 2 mL cryovials at minus 40 °C until testing (Corning Inc., Corning, NY, USA). All equipment used for sampling was cleaned and disinfected between pigs and uses. All samples were frozen and thawed once prior to the test. 

### 2.5. Determination of the Infectious Activities of ASFV

The infectious activities of the strains were determined by titration in PBLS (four wells for each tenfold dilution) by hemadsorption test (HAD). The results were examined by the presence of hemadsorption phenomenon after 5–7 days. The virus titres were calculated according to the method described by B.A. Kerber in I.P. Ashmarin’s modification and expressed in 50% hemadsorbing units per mL (HAU_50_/mL) [11]. 

### 2.6. Polymerase Chain Reaction (PCR) 

The viral DNA was extracted from all EDTA blood and saliva samples using the QIAmp_DNA Mini kit (QIAGEN, Hilden, Germany) according to the manufacturer’s instructions. Detection of ASFV genomic DNA was carried out according to the protocol described by Fernandez-Pinero et al. (2013) on Bio-Rad CFX 96 Real-Time Detection Systems (Bio-Rad, Hercules, CA, USA) [12]. Samples with Ct (cycle threshold) < 45.0 were considered as positive, while samples with no Ct value were considered as negative.

### 2.7. Detection of Anti-ASFV Antibodies

Serum samples were tested in duplicates using the INgezim PPA Compac solid-phase ELISA test kit (Ingenasa, Madrid, Spain) [13]. According to the kit instructions, the status of each tested serum was expressed using the coefficient of inhibition (x%). Oral fluid samples were tested using ID Screen^®^ African Swine Fever Oral Fluids Indirect expressed in OD_450_ [14].

### 2.8. Clinical Evaluation

The severity of the disease was assessed by a quantitative clinical score (CS) obtained by adding the values for the following eight clinical signs recorded on a daily basis, as detailed by Gallardo et al. (2015): fever parameters, anorexia, recumbency, skin hemorrhage or cyanosis, joint swelling, respiratory distress, ocular discharge, and digestive findings were assigned points on an ascending severity scale of 0–3. Pre-determined humane endpoints included a pig displaying severe signs of fever, anorexia, recumbence, respiratory distress and digestive signs for more than two consecutive days, or a total CS > 18 [15].

### 2.9. Statistical Analysis 

Statistical analysis of the results was performed using multifactor analysis. Differences between counts were considered significant at *p* < 0.05.

## 3. Results

**Figure 1:**
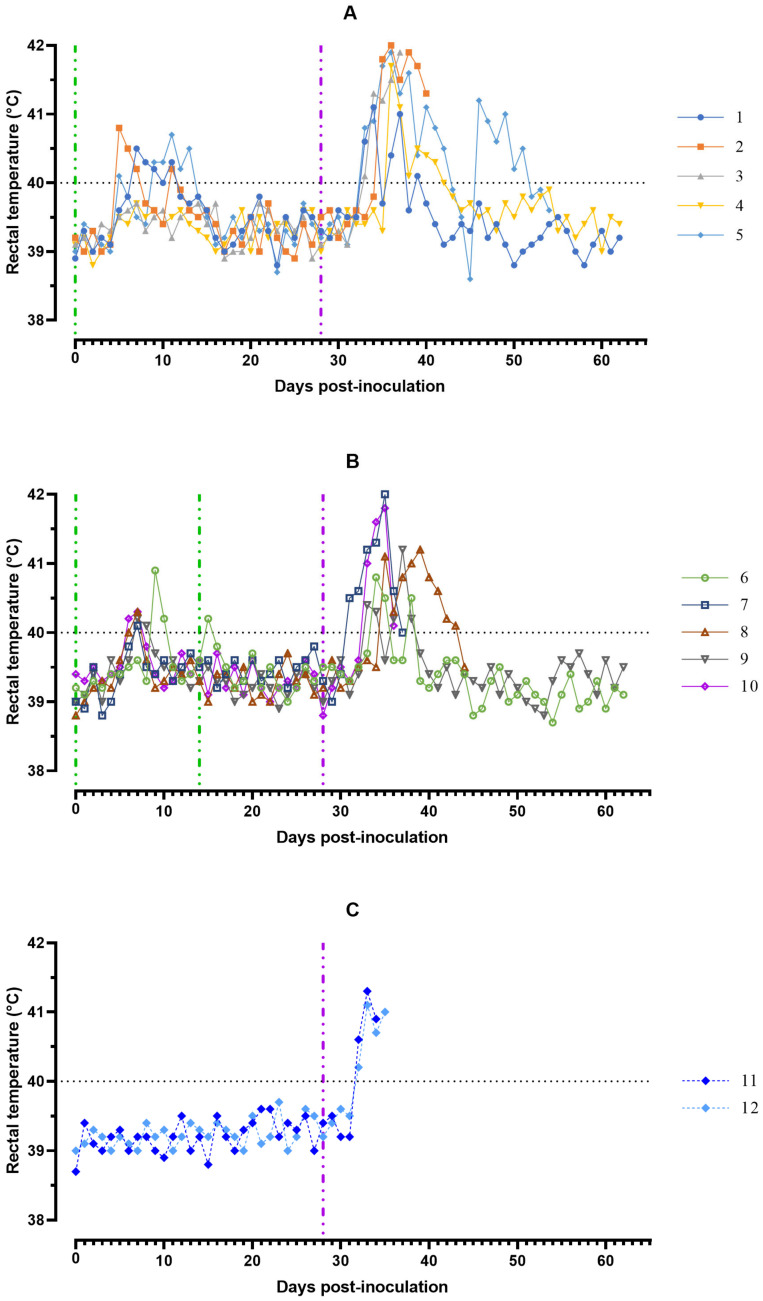
Kinetics of pig’s body temperature: (**A**) group #1 after inoculation with the ASFV-Katanga-350 and challenge on the 28th day with the ASFV-Stavropol 01/08; (**B**) group #2 after two inoculations with the ASFV-Katanga-350 and challenge on the 28th day with the ASFV-Stavropol 01/08; (**C**) group #3 after challenge with the ASFV-Stavropol 01/08. Each curve represents an individual animal’s values. Vertical dashed lines: green—days of inoculation of ASFV-Katanga-350, purple—challenge by the ASFV–Stavropol 01/08.

After inoculation on day 0 with ASFV-Katanga-350, 8 out of 10 pigs from groups #1 and 2 had an elevated body temperature of 40.1 to 40.9 °C during the period from 5 to 13 days ranging from 1 to 5 days. Together with hyperthermia, most of the animals showed a decrease in appetite and activity. After the second inoculation with the ASFV-Katanga-350, on day 14, the body temperature of pigs from group #2 was normal (Figure 1).

**Figure 2:**
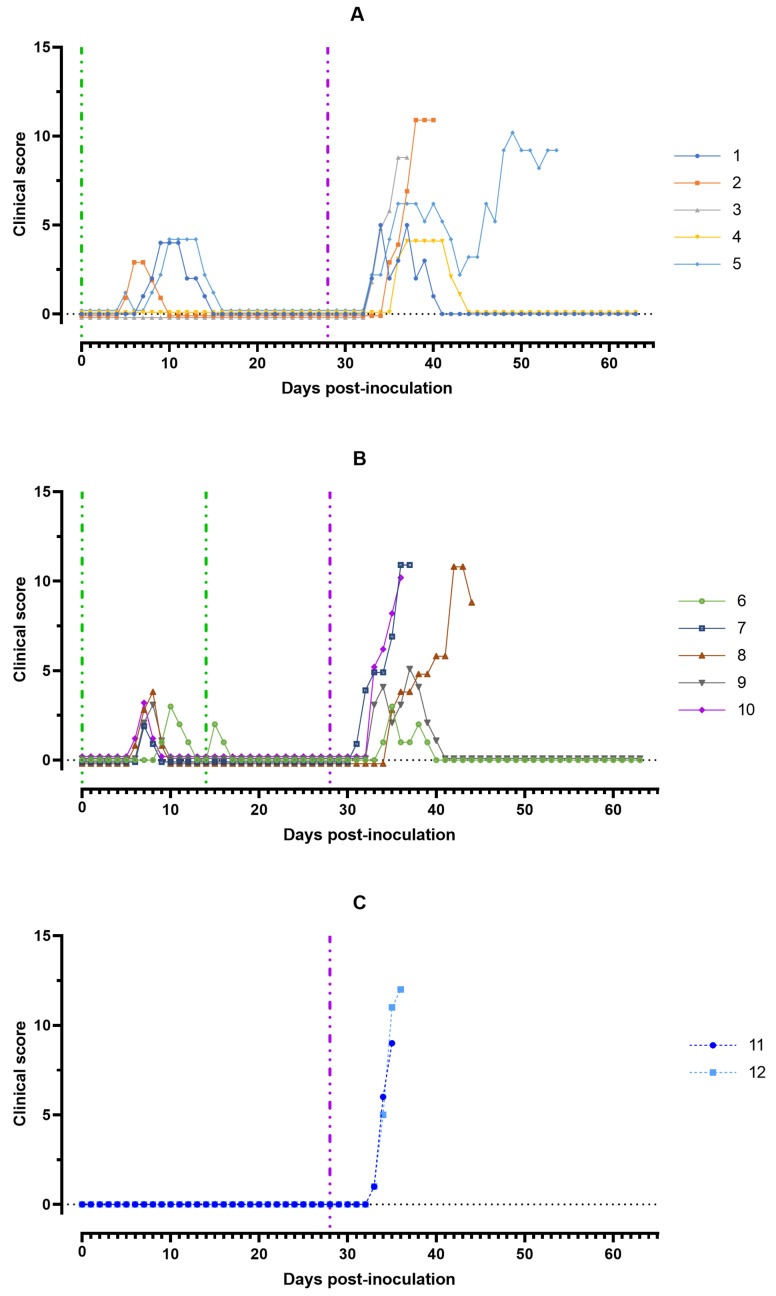
Clinical score of pigs: (**A**) group #1 after inoculation with the ASFV-Katanga-350 and challenge on the 28th day with the ASFV-Stavropol 01/08; (**B**) group #2 after two inoculations with the ASFV-Katanga-350 and challenge on the 28th day with the ASFV-Stavropol 01/08; (**C**) group #3 after challenge with the ASFV-Stavropol 01/08. Each curve represents an individual animal’s values. Vertical dashed lines: green—days of inoculation of ASFV-Katanga-350, purple —challenge by the ASFV–Stavropol 01/08.

The clinical score in domestic pigs increased from day 5 to 15 dpi to a maximum of 4 points mainly affecting liveliness, position, breathing, feed intake, and walk. By day 15 dpi all pigs from groups #1 and 2 clinically recovered. The indicators of clinical signs of ASF after challenge on the 28th day with the ASFV-Stavropol 01/08 reached 9–12 points in animals #2, 3, 5, 7, 8, 10, 11, and 12 from groups #1–3 that subsequently died and 3–5 points in animals #1, 4, 6, and 9 from groups #1 and 2 that survived. In challenged pigs, a decrease in appetite and activity was observed (Figure 2).

**Figure 3:**
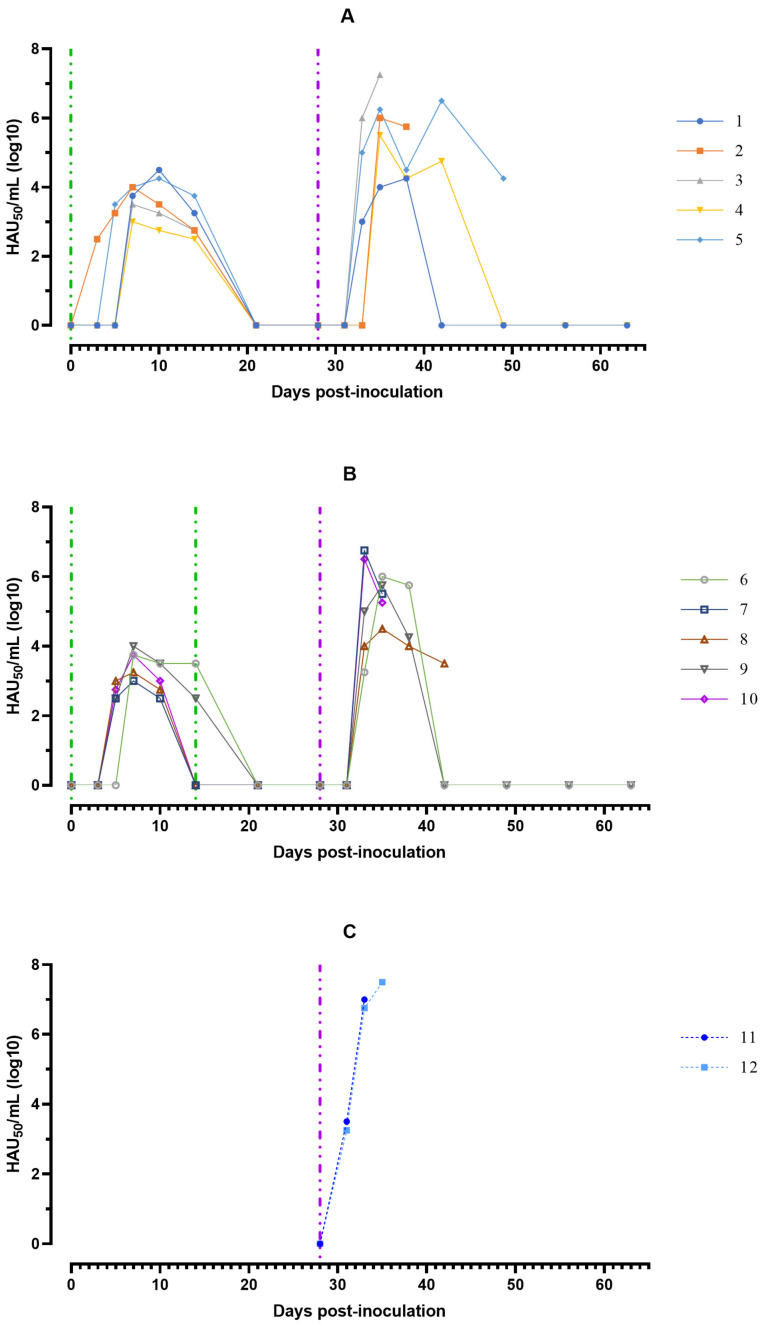
Virus titres in blood samples obtained from pigs: (**A**) group #1 after inoculation with the ASFV-Katanga-350 and challenge on the 28th day with the ASFV-Stavropol 01/08; (**B**) group #2 after two inoculations with the ASFV-Katanga-350 and challenge on the 28th day with the ASFV-Stavropol 01/08; (**C**) group #3 after challenge with the ASFV-Stavropol 01/08. Each bar represents an individual animal’s values. Vertical dashed lines: green—days of inoculation of ASFV-Katanga-350, purple—challenge by the ASFV–Stavropol 01/08.

The maximum values of viremia, 10^3.00^–10^4.50^ HAU_50_/mL, of animals from groups # 1 and 2 were recorded in the period from 7 to 10 days after the first inoculation of the ASFV-Katanga-350. The second inoculation of pigs from group #2 with ASFV-Katanga-350 did not lead to the accumulation of viremia. At 21 dpi, viremia, 10^1.75^–10^2.00^ HAU_50_/mL, was found only in two pigs out of ten, one in each group #1 and 2. At 28 dpi, in pigs from group #1 and 2 viremia was absent. After challenge (on the 28th day) with ASFV-Stavropol 01/08 the maximum viremia values, 10^7.00^–10^7.50^ HAU_50_/mL, were noted on the eve of death at 5–7 dpc in naïve animals #11 and 12 from group # 3. In surviving pigs # 1, 4, 6, and 9 from groups #1 and 2, viremia values on 7–10 dpc were lower—10^4.25^–10^6.00^ HAU_50_/mL. From 28 dpc ASFV was not detected in the blood of surviving pigs (Figure 3).

**Figure 4:**
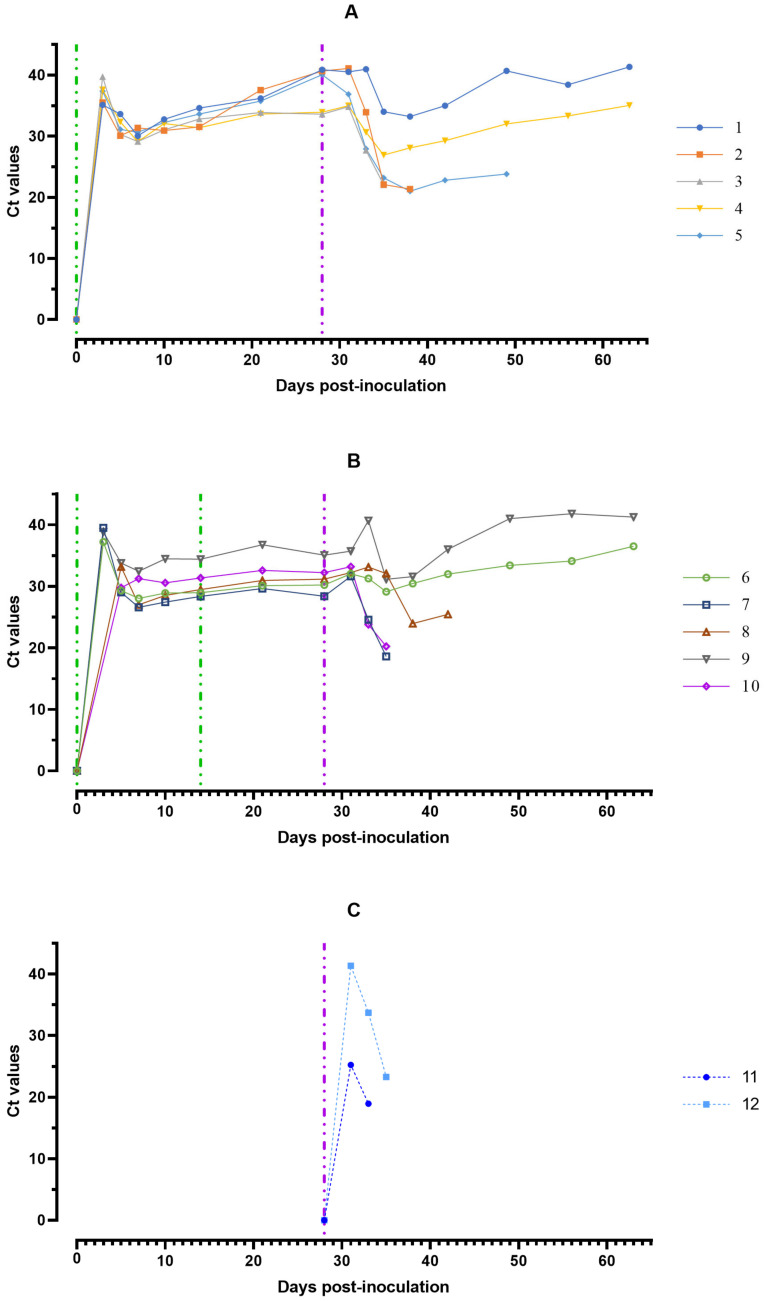
Values of ASFV-specific qPCR results of blood samples of pigs from 0 to 63 dpi: (**A**) group #1 after inoculation with the ASFV-Katanga-350 and challenge on the 28th day with the ASFV-Stavropol 01/08; (**B**) group #2 after two inoculations with the ASFV-Katanga-350 and challenge on the 28th day with the ASFV-Stavropol 01/08; (**C**) group #3 after challenge with the ASFV-Stavropol 01/08. Each bar represents an individual animal’s values. Vertical dashed lines: green—days of inoculation of ASFV-Katanga-350, purple—challenge by the ASFV–Stavropol 01/08. Results are displayed as Ct.

The minimum individual values of Ct in blood samples of eight pigs were marked by 7 dpi, in one by 5 and another one by 10 dpi. At 28 dpi, in pigs from group #1, Ct values were 33.58–40.86 (Ct = 37.81 ± 2.63), and in pigs from group #2 Ct—28.39–35.06 (Ct = 31.41 ± 2.22). The calculated value of Student’s *t*-test at a significance level of *p* < 0.05 turned out to be less than the critical one, so the differences in Ct in groups # 1 and 2 are not statistically significant (Figure 4).

**Figure 5:**
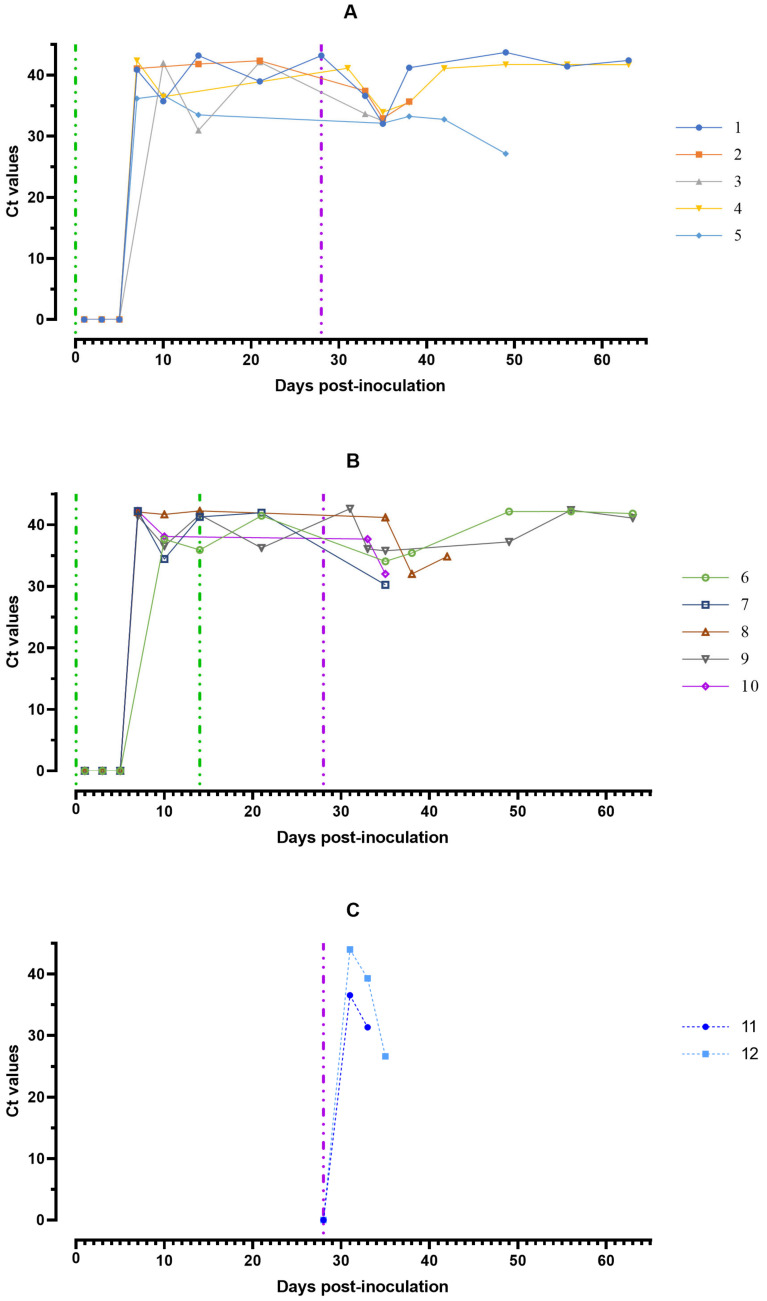
Values of ASFV-specific qPCR results of saliva samples of pigs from 0 to 63 dpi: (**A**) group #1 after inoculation with the ASFV-Katanga-350 and challenge on the 28th day with the ASFV-Stavropol 01/08; (**B**) group #2 after twice inoculation with the ASFV-Katanga-350 and challenge on the 28th day with the ASFV-Stavropol 01/08; (**C**) group #3 after challenge with the ASFV-Stavropol 01/08. Each curve represents the values of individual animals. Vertical dashed lines: green—days of inoculation of ASFV-Katanga-350, purple—challenge by the ASFV–Stavropol 01/08.

At 7–14 dpc ASFV-Stavropol 01/08 in all animals from groups #1 and 2 in blood and saliva samples, an increase of the concentration of viral DNA was noted (Figure 4 and Figure 5). By 21 dpc, Ct values were back to pre-infection levels. The values of the Ct were reached in blood/oral fluids samples (18.11–22.09/27.12–32.50) a day before euthanasia in naïve pigs from group #3 infected by ASFV–Stavropol 01/08 (Figure 4 and Figure 5).

**Figure 6:**
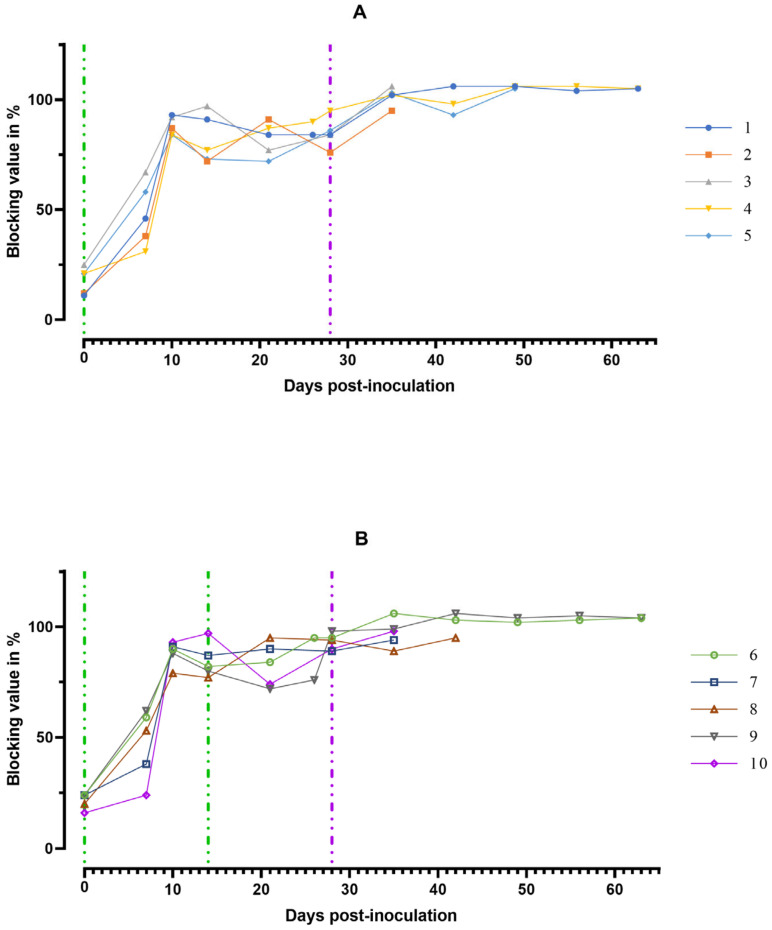
Individual kinetics of antibodies to ASFV in blood serum samples: (**A**) group #1 after inoculation with the ASFV-Katanga-350 and challenge on the 28th day with the ASFV-Stavropol 01/08; (**B**) group #2 after two inoculations with the ASFV-Katanga-350 and challenge on the 28th day with the ASFV-Stavropol 01/08. Each curve represents the values of individual animals. Vertical dashed lines: green—days of inoculation of ASFV-Katanga-350, purple—challenge by the ASFV–Stavropol 01/08.

From 7 dpi, the levels of virus-specific antibodies in blood serum samples were positive in 4 out of 10 pigs from groups #1 and 2. At 10 dpi, they reached maximum values and persisted until death or euthanasia of the animals (Figure 6). Infection at 28 dpi with ASFV-Stavropol 01/08 did not lead to a decrease in the level of virus-specific antibodies in both dead and surviving pigs (Figure 6).

Naïve pigs infected with the ASFV–Stavropol 01/08 strain were not tested for virus-specific antibodies as they all died between days 7 and 8 (data not shown).

In animals #2, 3, 5, 7, 8, 10, 11, and 12 from groups #1–3 the cyanosis of the tips of the ears, shortness of breath, labored breathing and/or coughing, as well as paresis and paralysis of the limbs or impaired walking were noted 1–2 days before death or euthanasia. Naïve animals #11 and 12 from group #3 died from an acute form of ASF on 7 and 8 dpc. Of the 10 animals of the experimental groups #1 and 2, four pigs, #2, 3, 7, and 10, died from acute form of ASF in the period from 9 to 12 dpc, and two pigs, #5 and 8, died from subacute form disease in the period from 17 to 27 dpc (Figure 1).

The multiplicity of inoculation of the ASFV-Katanga-350 did not affect the percentage of surviving animals after infection of pigs with the heterologous ASFV-Stavropol 01/08 (Figure 1).

**Figure 7:**
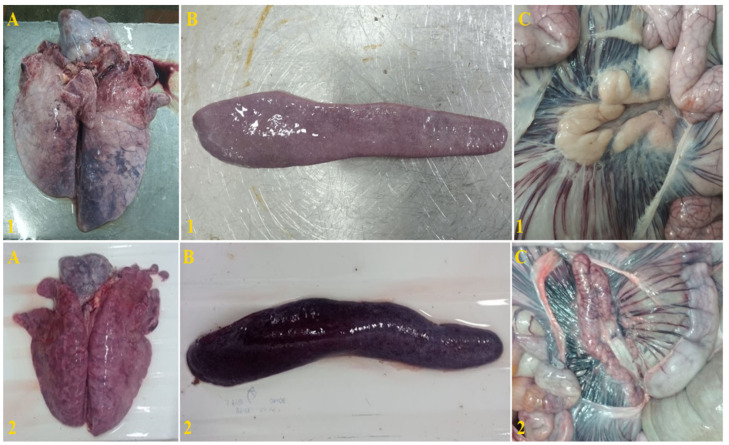
Lungs (**A**), spleen (**B**) and mesenteric lymph nodes (**C**) of pigs: #1 after inoculation with ASFV-Katanga-350, and followed by a challenge with ASFV-Stavropol 01/08; #2 after challenge with ASFV-Stavropol 01/08, respectively.

At autopsy of dead animals, pathoanatomical changes characteristic of acute and subacute forms of ASF were recorded. Serous-hemorrhagic and hemorrhagic lymphadenitis, the presence of hemorrhagic and serous-hemorrhagic exudate in the chest and abdominal areas, splenitis, congestive hyperemia of the lungs, liver and kidneys, pulmonary edema, as well as enteritis, in some cases colitis, and pneumonia. In the lungs of the surviving animals, after their euthanasia at 63 dpi, congestive hyperemia and pitting hemorrhages were detected. No pathomorphological changes were observed in other organs (Figure 7).

## 4. Discussion

The study of homologous and heterologous protection in ASF initially faces the choice of a basic method of classifying isolates and strains. Currently, genotyping of ASFV is based on an analysis of sequences from a few distinct genetic loci that demonstrate different levels of variability among diverse isolates. Standard methodologies include typing of the p72 capsid protein gene with concurrent analysis of the central variable region tandem repeats within the 9RL/B602L gene to provide intragenotypic resolution [16,17]. So far, a maximal variety of ASFV is found and described in Africa: 24 different genotypes, parted into four geographical clades [18]. However, it should be noted that the information based on genotyping of the ASFV p72 locus does not fully correlate with the available data on cross-protection, and as a result prediction of the efficacy of the candidate vaccines is difficult [7,19].

Seroimmunotypical classification of ASFV was established on the basis of serological typing by the hemadsorption inhibition assay (HAdI) in combination with an immunobiological test (protection of pigs against fatal infection by a virulent test virus after immunization with an attenuated virus strain of the homologous serotype) [20,21]. As a fast and accurate alternative for ASFV serogroup classification, CD2v/C-type lectin gene-based analysis has been proposed. It should be noted that CD2v gene sequencing with a following phylogenetic analysis could adequately predict ASFV strain serogroups and characterize their phenotypes [22,23]. 

Typing ASFV isolates into discrete seroimmunotypes is not necessarily resolved by conventional p72 genotyping, whereas samples ASFV of seroimmunotypes I, II, and IV are all genotype I (based on p72 genotype classification) [19,24]. In particular, ASFV strain 1455, which was derived from the Lisbon 60 isolate after being passaged up to 150 times in PBMC, protected against selected isolates circulating in Spain and Portugal in the 1960s, but did not protect against isolates obtained from the original outbreak in 1957 in Lisbon or from Katanga (now Democratic Republic of Congo, DRC). Later, it turned out that Katanga and Lisbon-57 by hemadsorption inhibition assay were serogroup I and Lisbon-60 was serogroup IV [19]. It is known that pigs infected with the non-pathogenic, non-HAD virus isolate OUR T88/3 or OUR T88/4 were protected from infection with the pathogenic HAD virus OUR T88/1 that was isolated on the same farm. The effectiveness of the protection was reduced when recovered pigs were challenged with the more distantly related Lisbon-57 isolate [25]. All isolates mentioned above belong to genotype I. However, the OUR T88/1, OUR T88/3, and OUR T88/4 isolates belong to serotype IV, according to the nucleotide analysis of CD2v/C-type protein genes, whereas the Lisbon-57 strain is a member of serotype I [19]. The death of pigs inoculated with the OUR T88/3 or OUR T88/4 isolate after being challenged with the strain, Lisbon-57, can be explained by the seroimmunological differences of these strains.

Another example, ASFV strains Rhodesia, Georgia 2007/1, and Stavropol 01/08 are assigned to seroimmunotype VIII, but according to genotyping, the Rhodesia strain is assigned to genotype VIII, while Georgia 2007/1 and Stavropol 01/08 are assigned by genotype II [19]. The attenuated strain RK-30 obtained by the passaging of the Rhodesia strain protected pigs from death, not only from the parental Rhodesia strain, but also from the ASFV-Stavropol 01/08 [21]. In our view, there is no contradiction in this. Genotype VIII ASFV isolates have also been detected in the neighboring countries of Zambia (formerly Rhodesia) and Mozambique. At the same time, genotype II isolates have been detected in Zambia, Zimbabwe, Mozambique, and Madagascar [26]. It can be assumed that numerous recombinant isolates can be formed in the sylvatic “cauldron” in Southeast Africa during ASFV transmission between warthogs and *Ornithodoros* ticks.

Despite their imperfections, LAVs available today confer a level of protection against experimental ASF infection far better than any other vaccine strategy so far tested. Cross-protective vaccines could be useful not only to protect a specific region against a single virus, but against many viruses from the same serotype or from different serotypes, thus covering endemic regions of Africa where up to nine seroimmunotypes have been described. The ability/inability of attenuated strains to form a certain level of protection against heterologous isolates needs theoretical background and experimental confirmation.

The history of research on obtaining a candidate live vaccine against ASF virus seroimmunotype I is mentionable. By selective passages in primary cultures of PBMC and PBLS of reference strain Lisbon-57 of the ASFV (seroimmunotype I), attenuated strain LK-111 was obtained. After the inoculation of strain LK-111 to pigs at a dose of 10^7.00^ HAU_50_, protection against a subsequent intramuscular challenge with virulent strain Lisbon-57 at a dose of 10^4.00^ HAU_50_ was formed in only 50–70% of the immunized pigs. Because of the low protection, prolonged viremia after inoculation to pigs strain LK-111 was not recommended for the development of vaccine preparations. Further, the virulent hemadsorbing strain Katanga-105 isolated in Zaire (DRC) was used as a starting point. To attenuate the Katanga-105 strain, the virus was passed on the PBMC culture by limiting virus dilutions with the selection of clones with low hemadsorbing activity. Nonhemadsorbing virus variant Kc-160 was obtained when administered intramuscularly to pigs at a dose of 10^7.50^ TCID_50_ (50% Tissue Culture Infectious Dose), causing a weak or moderate clinical response in 75–80% of the pigs. Viremia lasted no more than 28 days. On day 14, 80–100% of the pigs formed a resistance to intramuscular infection of 10^4.00^ HAU_50_ of strain Lisbon-57. After 30 days, the virus was not detected in the blood of most of the pigs. To assess the effects of using the Kc-160 variant on pigs with a lowered immune status, a group of pigs with a low level of white blood cells and with symptoms of gastroenteritis, bronchopneumonia, and arthritis was formed. After intramuscular inoculation of the Kc-160 variant at a dose of 10^6.50^ TCID_50_, 20% of these pigs died on days 9–14 [21]

As a result of passaging in the PBMC culture of virulent ASFV strain Katanga of seroimmunotype I, attenuated hemadsorbing strain Katanga-350 was obtained [21]. ASFV-Katanga-350 is a low virulent strain that causes very weak or unapparent clinical symptoms in domestic pigs. Based on confirmed moderate levels of viremia and the manifestation of clinical signs, ASFV-Katanga-350 can be considered as a basis for the development of a more advanced live recombinant vaccine against the ASFV seroimmunotype I [8].

According to previous studies, all animals inoculated with an attenuated strain developed a high antibody response after a week post infection, which was maintained until the end of the experiment [15,25,27]. We found similar results at 10 dpi, at which the levels of virus-specific antibodies reached maximum values and persisted until death or euthanasia of the animals. A high level of virus-specific antibodies in the blood sera until the end of the experiment, apparently, suggests a persistence of the virus in pigs. Although in accordance with the results of Real-Time PCR, the detection of viremia in surviving pigs showed its absence in the blood. We did not observe a close relationship between the presence of circulating virus-specific antibodies and protection. In our experience, the presence of long viremia or virus persistence indicates insufficient effectiveness of the formed protective immunity. The challenge of virulent Lisbon-57 virus following ASFV-Katanga-350 inoculation acts to boost the immune response, and this might be required for inducing cross-protective immunity to ASFV-Stavropol 01/08. After one or two inoculations with the ASFV-Katanga-350, challenge with the ASFV-Stavropol 01/08 did not protect animals from the disease in case of subsequent infection with heterologous ASFV-Stavropol 01/08, but 40% of the pigs protected from death.

These results differ slightly from our studies of homologous and heterologous protection induced by attenuated strains III and IV of seroimmunotypes MK-200 and FK-32/135 with respect to the corresponding virulent strains, Mozambique-78 and France-32. As a result of our earlier work, we observed 100% protection against homologous strains and 0% protection against heterologous strains [28]. Note that based on the EP402R (CD2v) gene phylogenetic analysis, strains I, II, III, V, and VIII of serotypes belong to one branch of the phylogram, whereas strains III and VIII form one cluster, while strains I and II of serotypes form separate neighboring clusters [29]. It is possible that this division into clusters and genetic, and probably antigenic, affinity of strains I, II, III, and VIII seroimmunotypes resulted in the formation of partial protection against the death of ASFV-Katanga-350 (I serotype)-inoculated pigs subsequently challenged with heterotype ASFV-Stavropol 01/08 (VIII serotype). This assumption can be experimentally investigated using attenuated ASFV III seroimmunotype and virulent ASFV-Stavropol 01/08 VIII serotype. In summary, inoculation of pigs with the Katanga-350 strain did not protect animals from the disease in the case of the subsequent challenged strain Stavropol 01/08. Moreover, the surviving animals showed no gross pathomorphological changes or the presence of an infectious virus in the blood at 35 days’ post challenging. Our research is relevant when designing LAVs against ASFV, since on most occasions they limit their protection against the parental virulent strain but not against heterologous strains.

## Data Availability

The data presented in this study are available on request from the corresponding author.
